# Enabling visually impaired people to learn three-dimensional tactile graphics with a 3DOF haptic mouse

**DOI:** 10.1186/s12984-021-00935-y

**Published:** 2021-09-25

**Authors:** Mariacarla Memeo, Marco Jacono, Giulio Sandini, Luca Brayda

**Affiliations:** 1 Present Address: Robotics, Brain and Cognitive Sciences Department Now with Cognition, Motion and Cognitive Science (CMON) Unit, Fondazione Istituto Italiano di Tecnologia, Via Enrico Melen 83, Genoa, Italy; 2 Robotics, Brain and Cognitive Sciences Department, Fondazione Istituto Italiano di Tecnologia, Via Enrico Melen 83, Genoa, Italy; 3 Acoesis srl, Via Enrico Melen 83, Genoa, Italy; 4 University of Genoa, Genoa, Italy

**Keywords:** Object recognition, Haptics, Visual impairment, Workload, Geometry

## Abstract

**Background:**

In this work, we present a novel sensory substitution system that enables to learn three dimensional digital information via touch when vision is unavailable. The system is based on a mouse-shaped device, designed to jointly perceive, with one finger only, local tactile height and inclination cues of arbitrary scalar fields. The device hosts a tactile actuator with three degrees of freedom: elevation, roll and pitch. The actuator approximates the tactile interaction with a plane tangential to the contact point between the finger and the field. Spatial information can therefore be mentally constructed by integrating local and global tactile cues: the actuator provides local cues, whereas proprioception associated with the mouse motion provides the global cues.

**Methods:**

The efficacy of the system is measured by a virtual/real object-matching task. Twenty-four gender and age-matched participants (one blind and one blindfolded sighted group) matched a tactile dictionary of virtual objects with their 3D-printed solid version. The exploration of the virtual objects happened in three conditions, i.e., with isolated or combined height and inclination cues. We investigated the performance and the mental cost of approximating virtual objects in these tactile conditions.

**Results:**

In both groups, elevation and inclination cues were sufficient to recognize the tactile dictionary, but their combination worked at best. The presence of elevation decreased a subjective estimate of mental effort. Interestingly, only visually impaired participants were aware of their performance and were able to predict it.

**Conclusions:**

The proposed technology could facilitate the learning of science, engineering and mathematics in absence of vision, being also an industrial low-cost solution to make graphical user interfaces accessible for people with vision loss.

## Background

*Object exploration in absence of vision* Constructing mental representations of real objects is generally achieved with both visual and tactile cues.

Those who use touch to identify object features, i.e., blind and visually impaired persons, develop autonomous or guided strategies and gain experience, in principle different from sighted subjects [1]. Whether or not people with vision loss construct cognitive maps similarly to sighted persons is still a matter of debate [2]. When objects are to be haptically manipulated with two hands, blind participants generally outperform their sighted peers [3]. The same holds in recognition of basic geometrical shapes in raised-outline drawings. This trend is confirmed also if shapes are presented in an unusual orientation or with distorted contours, thus with no prototypical objects [4]. However, vision loss seems not to be a factor when discriminating 3D grating orientation [5]. If object matching is done with hand-sized unfamiliar objects, the accuracy is still similar between groups [6–8].

*Haptic cues for cognitive mapping* The overall ongoing debate about cognitive mapping with touch motivated several works to understand the physical features that are necessary for humans to make sense of an object, namely shape and size [9].

Proprioception gives information about global features of solids, such as size, orientation and shape [10]. Moreover, proprioception helps in judgements based on local tactile cues [11, 12].

Maps in 2.5D, in which height profiles mimic 3D objects, are generally harder to understand [13, 14]. However, when casting the problem of understanding objects in a virtual setup, height profiles are quite effective [15] and especially when blind people are involved, elevation cues appear fundamental [16]. Combining proprioceptive cues and minimal tactile feedback with elevation cues is effective in displaying virtual multi-level objects [17], both in blind and sighted participants, with underpinnings found at the neurophysiological level [18, 19].

Since it is spontaneously chosen among other strategies [20], the preferred technique to perceive a shape feature is to slide a finger across an object surface [21]. A quantitative method to investigate haptic shape identification was introduced in [22]. Mathematically well-defined objects were explored and authors found that curvature was the most important feature to discriminate shapes. How to render curvature to facilitate the understanding of tactile objects is therefore important. In [23, 24] the relative contribution of geometrical descriptors of curvature in a shape discrimination task was explored. Inclination cues were found to be dominant over height cues.

*How many hands? How many fingers?* Another important aspect is the availability of limbs, i.e., the number of hands and fingers, as it drives the design of interfaces.

Against what it may sound obvious, using all available fingers and hands to perceive object features is not always necessary: one hand is preferred to reach better precision in stimuli discrimination [25]. Similarly, the comparison of curved profiles is more efficient with one hand [26]. As well, the main exploratory procedure for perceiving shapes, i.e. contour following, is more frequently performed with one finger only [27]. The use of two fingers, instead of one, shows no improvement in perceiving outlines of 2D shapes and pictures [28, 29]. Nor increasing the number of contact areas improves the performance in identifying real objects, but increasing tactile information per unit area does [30]. When exploring unknown surfaces, both normal and tangential forces are important: although tangential forces give sufficient cues to perform contour following tasks and discover geometrical elements [31], they are not necessary to understand elements larger than the fingerpad area, for which both inclination and elevation cues seem sufficient [32]. In fact, Frisoli and colleagues [33] showed that it is plausible to couple freehand exploration with partial haptic feedback on one finger only, to allow exploration and understanding of tactile virtual objects.

*Haptic technologies using one finger only* Motivated by the possibility to make sense of an object with a reduced set of features and limbs, many technologies were proposed to virtually render a variety of physical properties, mainly in setups where the objects cannot be seen.

The Haptic Tabletop Puck [34], the VTPlayer [35], among others ([33, 36–38] offer comprehensive reviews) are solutions merging cutaneous cues on one finger with kinesthetic cues, to perceive simple geometrical sketches in a partial or total absence of vision. Laterotactile displays [39] convey the illusion of exploring height profiles on a flat surface by generating lateral skin deformation. An overall agreement is that tactile mice might be useful in educational contexts: these have been coupled with audio to explore graphics [40] or to teach science [41]. Indeed, the VT-Player [42] provides tactual information about shapes and edges of a map with metallic pins of two Braille cells. The Virtouch Mouse [11] is a tactile mouse, coupled with a navigation screen that allows to recognize and draw shapes, diagrams and pictures.

However, all mentioned solutions mainly present information in dynamic conditions: when the finger is steady, no cue is given. An exception is the Tactus system proposed by Ziat [43], which allows exploring the environment with two hands to integrate local information on one finger of one hand (resting on a pin array matrix) with global information coming from proprioceptive cues of the other hand (manipulating a stylus). This allows to learn virtual images but keeps separated the process of exploring virtual objects and acquiring tactile information.

*Combining 3DOF tactile cues with proprioceptive cues* In summary, previous studies seem to hint that it is possible to deliver to one finger approximated three-dimensional virtual objects, at the condition that the hand is free to move to somewhat compensate for the lack of richness of the cutaneous feedback. The technique to present virtual surfaces appears to modulate the role of visual disability in building mental representations of real objects.

In this work, we studied if and how it is effective to render geometrical solids on one finger with a 3DOF actuator, employing a mouse-shaped device. Local spatial cues of the solid surface were approximated by the actuator, which stimulated one fingerpad: we studied two cues (elevation and inclination), given alone or in combination, at the contact point between the finger and the solid. Global spatial cues were instead actively sought by the hand-arm motion, while the hand rested on the mouse. The efficacy of the device in representing objects was assessed with a matching task, where virtual objects, actively constructed by using the mouse, were matched to a dictionary of real objects.

We also investigated whether the ability to match real to virtual objects depended on the kind and amount of geometrical descriptors (first independent variable) and vision loss (second independent variable). We hypothesised that if information from elevation and inclination provide complementary cues, then matching abilities should perform at best when both cues are present. Against what occurs with real objects, we also hypothesized that visual impairment would affect performance, taking into account that unfamiliar three-dimensional objects cannot be manipulated like real objects. Additionally, since the process of interaction and learning of a new environment cannot discard the mental resources associated with information display [44], we measured if mental workload [19, 45] and self-evaluation of performance [46] could also depend on the geometrical descriptors and vision loss.

## The TActile MOuse 3

### Concept

The TActile MOuse 3 (TAMO3) is a novel tactile mouse, able to deliver graphical content by approximating a surface with its elementary geometrical descriptors of the first two orders [47]. In particular, elevation (0*th* order from now on) and inclination (1*st* order from now on) can be rendered alone or in combination. Therefore, TAMO3 approximates real touch, by reproducing phalanx movements and normal fingertip deformations, respectively, with elevation and inclination cues. Tangential forces are not rendered, since the working principle is to display geometrical surfaces that do not require finer tactile abilities, or carry information via texture or do not elicit illusions [48].

**Fig. 1:**
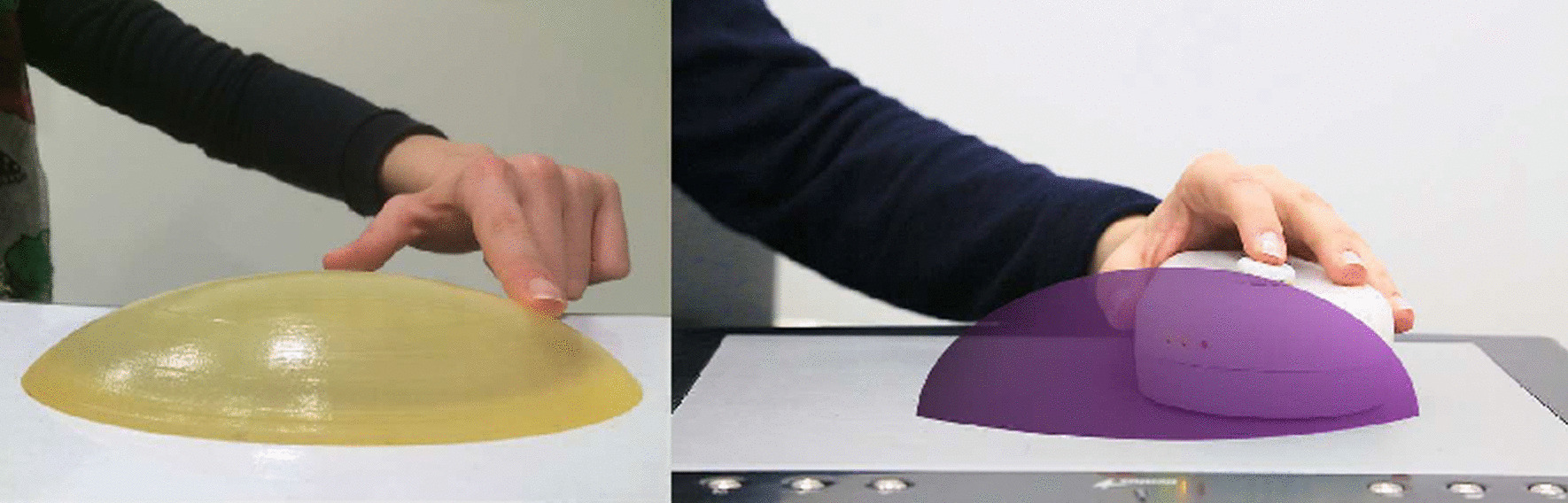
Exploration of a real object with a bare finger sliding on a real surface (left). The correspondent virtual object tactually rendered with TAMO3 (right)

The goal of using a mouse is to approximate the motion of a hand and arm actively exploring the shape of the equivalent real object. The principle is shown in Fig. 1. The mouse has a tactor (i.e., the end effector of an actuator capable of stimulating the sense of touch) that renders three tactile degrees of freedom, in each point of the virtual object. The stimulation is designed to be felt by a single finger passively resting on the tactor. The haptic feedback on the hand is composed of the kinesthetic feedback, rendered on the finger phalanxes, and by the tactile feedback rendered on the fingertip. Globally, the indentations on the fingertip during the active exploration induces dynamic changes in contact forces and area [49], which close the sensory-motor loop and can induce the user to plan and execute the mouse motion.

The substantial difference between TAMO3 and point-like end-effectors is the static rendering of inclination. With TAMO3, rendering the gradient of a three-dimensional surface does not require hand or arm motion, but can be inferred by tactile cues alone. Thus, the design goal was to elicit an understanding of object slopes with tactile and haptic cues close to those experienced during the exploration of a real object with a bare finger.

**Fig. 2:**
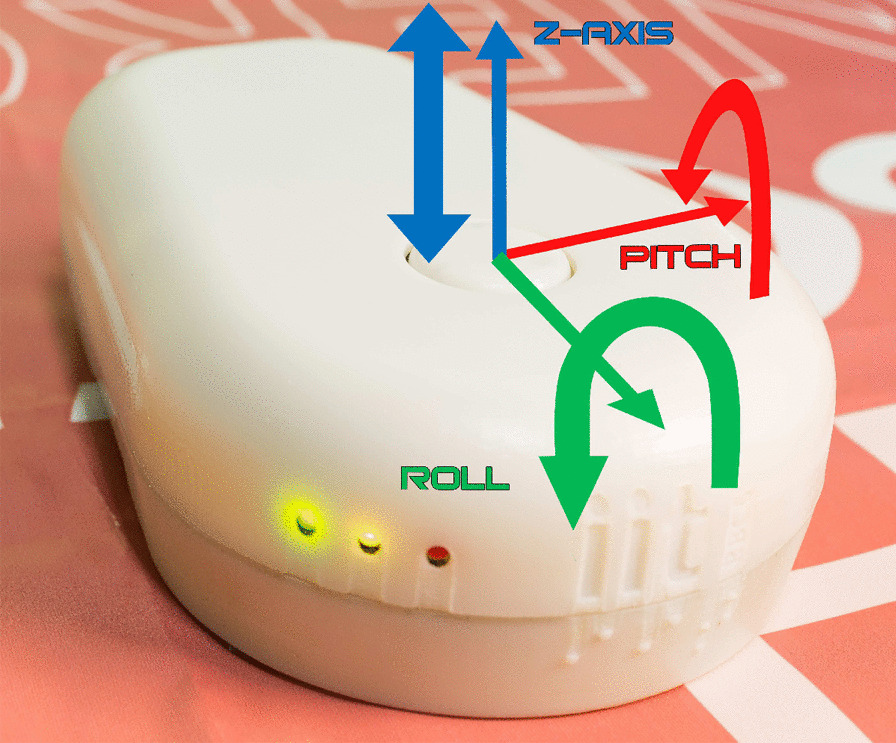
The TActile MOuse 3 (TAMO3) is a portable mouse able to produce tactile feedback on one finger only, employing a flat end effector (tactor). The tactile feedback aims at mimicking real touch and is provided through three degrees of freedom: elevation, roll and pitch. A video presenting the functioning of TAMO3 is available here: https://wesharescience.com/pin/10188

### Mechanics

TAMO3 presents a tactile display and a 3DoF Revolute-Revolute-Spherical (RRS) motion platform supporting rotation and translational movement [50]. It allows motion of the tactile end effector, i.e., the tactor, across the Z-axis (therefore eliciting elevation cues) as well as around the two axes forming the plane perpendicular to the Z-axis (roll and pitch cues). The tactor and the axes are shown in Fig. 2. The tactor is a moving disk, with a diameter of 20mm. Its motion is controlled by three independent servomotors which are connected to three pushing rods nestled in the lower surface of the tactor in three points, 120 degrees far apart from each other. The tactor motion can be assimilated to the stationary swashplate of a helicopter with cyclic/collective pitch mixing control, since the yaw cue is absent [51]. Both the tactor and the mouse external cover are built out of a 3D-printed ’verowhite’ resin.

**Fig. 3:**
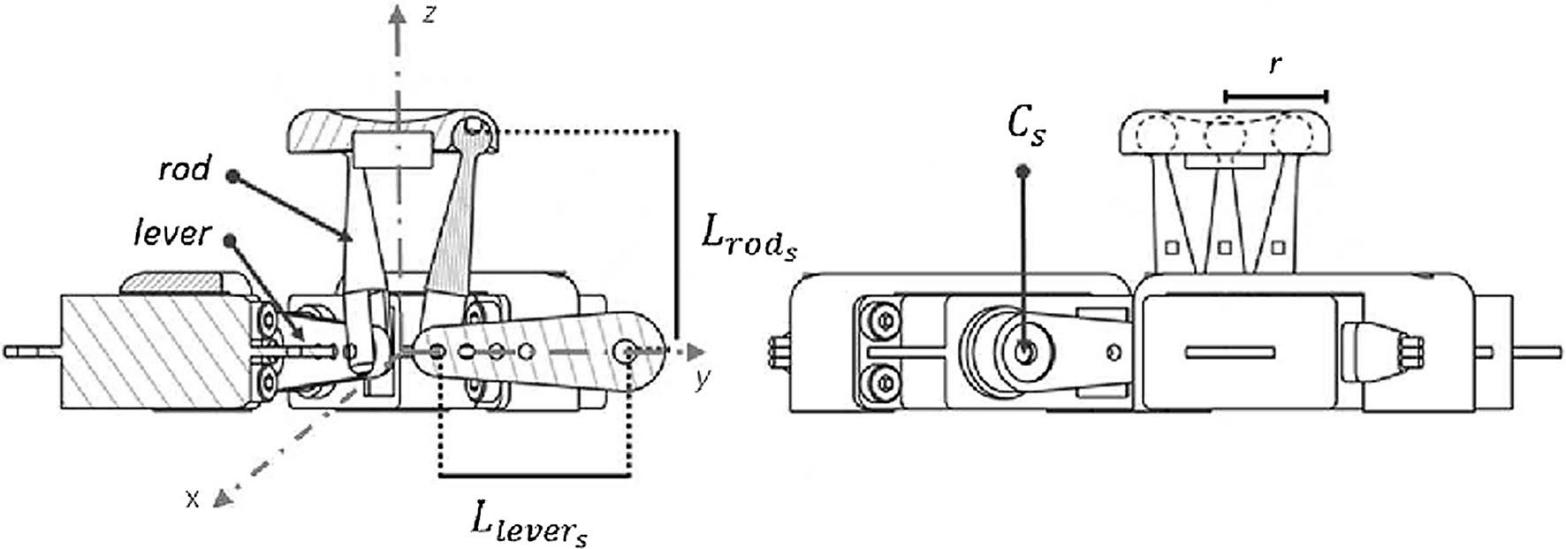
Sections of TAMO3 mechanical components with emphasis on the kinematic chain. The levers attached to the servos have length $L_{lever_{s}}$. They transmit the motion to three metallic rods that have length $L_{rod_{s}}$. $\hbox {C}_{{s}}$ is the coordinate array of lever centres of rotation and *r* is the tactor radius

The three Hitec HS-5056MG servomotors (see the “Appendix” for details) are controlled by three signals with pulse width modulation at a frequency of 200 Hz, having a width that varies from 1 to 3 ms (PWM signal). The movements of the servomotors are transmitted to the tactor thanks to three rods fastened to their levers through a linchpin and to the tactor base through a spherical joint, see Fig. 3. The TAMO3 refreshes the tactor elevation and inclination every 20 ms, regardless of how fast the user moves the mouse.

### Kinematic model

**Fig. 4:**
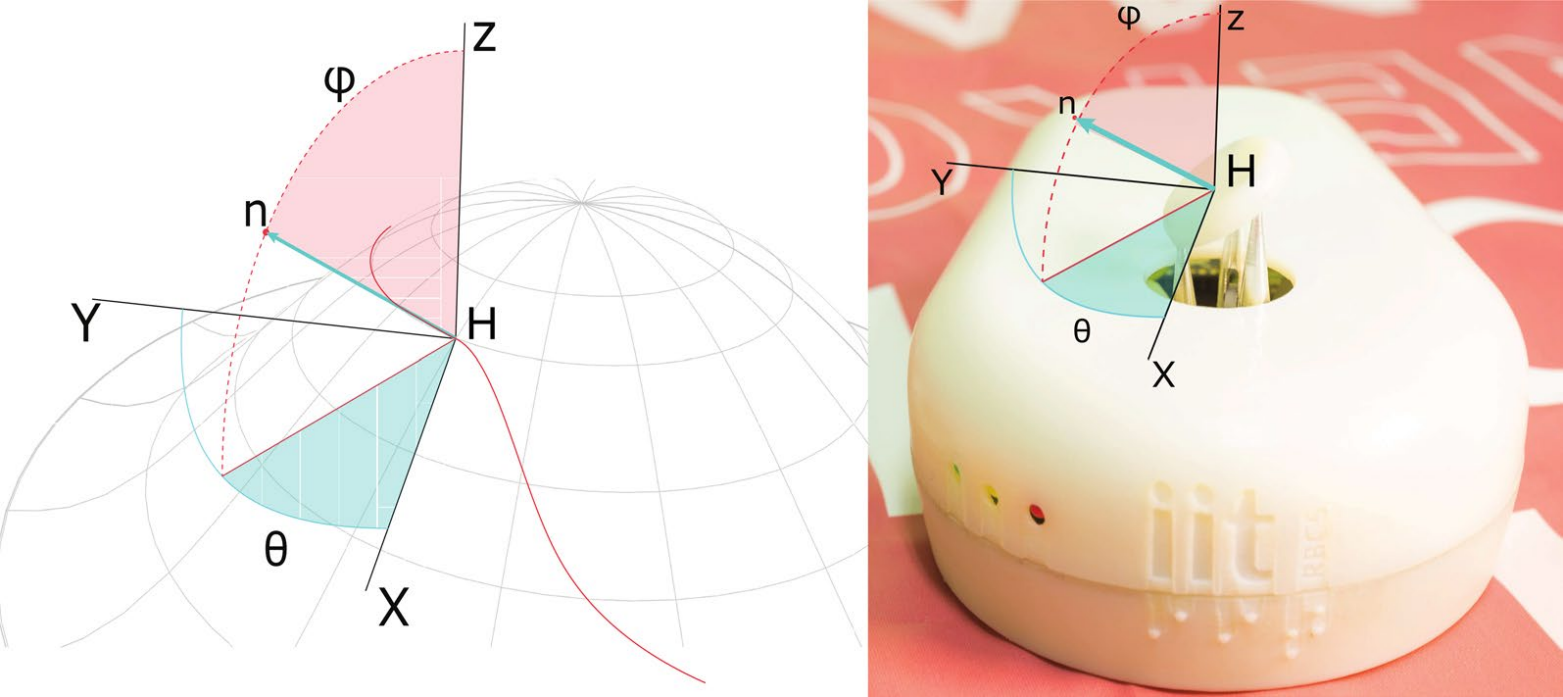
The displacement of the lever is expressed in terms of spherical coordinates. $\varphi$ is the *elevation* angle, $\theta$ represents the *azimuth* angle and *n* the unitary vector normal to the surface. Left: digital rendering of an arbitrary scalar field. Right: Local representation of the scalar field displayed with TAMO3

The kinematic model returns the values of the angles of the servomotors having in input tactor elevation (z-axis variable shown in Fig. 2) and the three components of the normal vector of the tactor expressed in spherical coordinates (see Fig. 4). Starting from the geometric disposition and physical dimension of the components of TAMO3, it is possible to calculate the coordinates of the center of rotation, $C_{s}$, for the lever of each servo, with respect to the origin of the global coordinate system showed in Fig. 3 in gray dashed lines:

$$\begin{aligned} C_{s}&= \begin{bmatrix} (L_{lever_{s}} + r)\ cos\alpha _{s}\\ (L_{lever_{s}} + r)\ sin\alpha _{s}\\ 0 \end{bmatrix} \end{aligned}$$

where $L_{lever_{s}}$ is the length of the lever, *r* the radius of the tactor, $\alpha$ the orientation of the lever on the *xy* plane and *s* = 1,2,3 the index of the three servos. The global coordinate system is expressed in Cartesian coordinates.

Each point of a virtual surface, expressed as a scalar field, can be uniquely identified with an elevation vector and a normal vector (see Fig. 4). These two parameters can be expressed as:

$$\begin{aligned} h = \begin{bmatrix} 0 \\ 0 \\ H \end{bmatrix} \qquad \qquad n = \begin{bmatrix} sin\varphi \ cos\theta \\ sin\varphi \ sin\theta \\ cos\varphi \end{bmatrix} \end{aligned}$$

where *h* is the array containing the height of tactor respect to the origin shown in Fig. 3; *n* the array with the components of the vector normal to the tactor and the application point at the center of the tactor; $\varphi$ the *elevation* angle, i.e., the angle formed by the positive semiaxis of z and the ray outgoing from the origin and passing through *n*; $\theta$ the *azimuth* angle, i.e., the angle formed by the positive semiaxis of x and the ray on the *xy* plane outgoing from the origin and passing through the projection of *n* on the same plane (see Fig. 4 for more details). Given the position of the versors pointing towards servos on the *xy* plane, $u_{s}$, the parametric equation to find $t_{s}$ in order to set the distance between *h(t)* and H can be solved, i.e. the position of the end-effector of the rod can be found.

$$\begin{aligned} u_{s}&= \Vert (-C_{s} \times (h + C_{s}))\times n \Vert \\ t_{s}&= \sqrt{\dfrac{r^{2}}{\sum \limits _{j} u_{s}^{2}}}\\ \end{aligned}$$

where *r* is the radius of the tactor.

For each servo, the final position of the rod in three dimensions, $\textit{S}_{s}$, is found with the equation:

$$\begin{aligned} S_{s}&= h + u_{s}*t_{s} \end{aligned}$$

In order to calculate the angles of the levers, a new set of coordinate system [*vx vy vz*] and its relative rotation matrix $\textit{R}_{s}$ is created:

$$\begin{aligned} vx&= \Vert C_{s} \Vert \\ vz&= \Vert (h + C_{s}) \times vx) \Vert \\ vy&= \Vert vz \times vx \Vert \\ R_{s}&= \begin{bmatrix} vx^\intercal&vy^\intercal&vz^\intercal \end{bmatrix} \end{aligned}$$

The coordinates of the rod are transformed from 3D in 2D with the rototranslation:

$$\begin{aligned} s_{s}&= (S_{s} - C_{s}) * R_{s} \end{aligned}$$

and zero value is assigned to the third component.

Then it is possible to obtain the values of the angles of lever, $\delta _{lever_{s}}$, expressed in radiants:

$$\begin{aligned} \delta _{lever_{s}} =&\Bigg (\dfrac{sin(py)}{cos(px)}\Bigg )^{-1} \\&- \Bigg (\dfrac{sin(L_{rod_{s}}*b_{s})}{cos(L_{lever_{s}}+L_{rod_{s}}*a_{s})}\Bigg )^{-1} \end{aligned}$$

where $px_{s}$ and $py_{s}$ are respectively the first and second components of $s_{s}$ in 2D and *a*, *b* are parameters depending on physical dimensions of the components of TAMO3.

### Invariance of the tactor inclination to hand/wrist rotation

Since we concentrate the tactile feedback on one finger only, our idea is to enrich it by approximating the tactile cues related to freehand exploration. In particular, when a certain fingertip area is in contact with a surface and the hand rotates, the location of the fingertip area changes according to the amount of hand rotation. Since such rotation can also be unintentional, the inclination of the surface can be misunderstood if the related inclination of the tactor is not aligned with the user’s body. Therefore, the tactor inclination in each point of the surface must be invariant to hand rotations. In other words, the mouse has to compensate for rotations of the hand/wrist by simulating the missing degree of freedom (yaw). In general, tactile devices aimed at stimulation in virtual setups lack this feature.

The main difficulty of updating the tactile feedback according to wrist orientation consists in the time required to measure the angular position of the wrist and, consequently generating adequate tactile feedback. To the best of our knowledge, this device is the first attempt to face this issue.

To avoid misunderstanding of a constantly updated inclination of the tactor (cause by wrist motion), the refresh of tactile feedback should occur in the range of 30–40 ms [52]. When the wrist/hand rotates, the TAMO3 refreshes the tactor orientation every 33 ms. This feature was not included in the experiments of this manuscript.

### Absolute positioning system

The position of the tactile mouse is detected using a commercial graphic tablet (Genius PenSketch M912) connected via USB to a computer (Dell Precision M64000) hosting Windows 7. The tablet worked at 1920 x 1200 pixels, spanned on a working area of 228 x 305 mm. The dimensions of the computer display and the tablet were normalized in order to make an exact correspondence between the movement of the mouse done by the user and the relative movement of the pointer on the screen. The tablet sends the current absolute XY position of the tactor to the computer via USB at a frequency of 50 Hz. Two coils similar to those built in the tablet pen were integrated on the board of the TAMO3, to retrieve the hand orientation: the two coils were alternatively activated so that the Windows mouse driver could see two ’virtual’ pen points. The segment joining the two point was equal to the mouse orientation.

## Experiment: virtual objects match

### Materials and methods

*Participants* The sample included blindfolded sighted (BS) and visually impaired (VI) participants. BS participants were 12 volunteers: 6 females, 24–36 years, $29.1 \pm {4.2}$ sd. The VI sample matched in gender and age the sample of BS participants (12 volunteers: 6 females, 19–29 years, $24.4 \pm {3.3}$ sd). According to the World Health Organization classification, 8 of them were blind belonging to the 4*th* category and 4 were blind of the 3*rd* category meaning that their acuity ranged from 1/60 to 1/20 [53]. Nine participants of the VI sample were congenitally blind and three lost their sight at puberty. Informed consent was obtained from all participants and protocols complied with the Declaration of Helsinki.

All participants were naïve to the task, reported to be right-handed and had no scars on the fingertip used for the experiment.

**Fig. 5:**
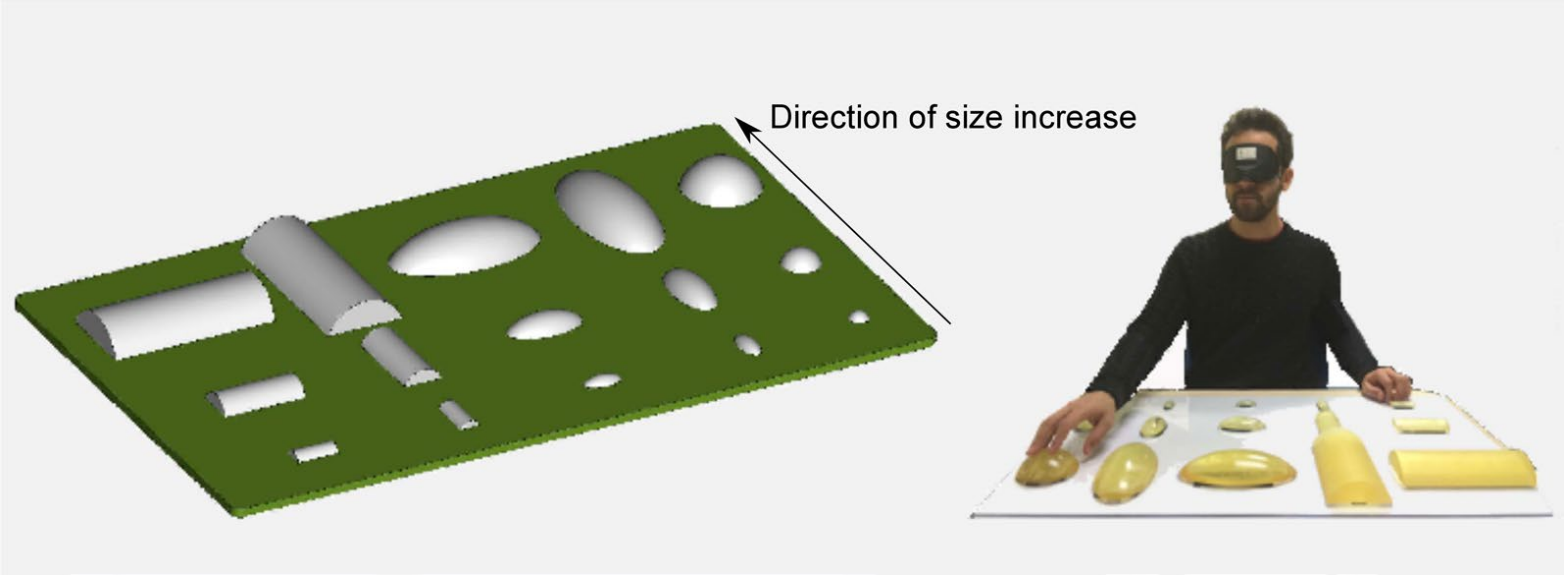
Left: CAD model of the Tactile Dictionary used as verification setup. It included five main solids: one sphere, two cylinders and two ellipses. The latter two are arranged in two orientations. The main parameters of the solids were 50 mm (equal to the diameter of the sphere, the smallest side of the cylinder and the minor axis of the ellipse) and 100 mm (equal to the largest side of the cylinder and the major axis of the ellipses). The height of all objects was 18 mm. Right: the real scenario in which one participant is exploring the Tactile Dictionary

*Setup* The participants were asked to explore five virtual objects using TAMO3. A Tactile Dictionary of fifteen real 3D-printed solid objects served as a verification setup (see Fig. 5). They were arranged both transversely and long ways with respect to participants’ bodies and were attached with Velcro®to an 800 × 500 mm Plexiglas panel.

Those five objects were replicated exactly doubled and halved in volume: thus the upper row in Fig. 5 (left) is composed of *over size objects*, the middle of *matched size objects*, the lower of *under size objects*.

**Fig. 6:**
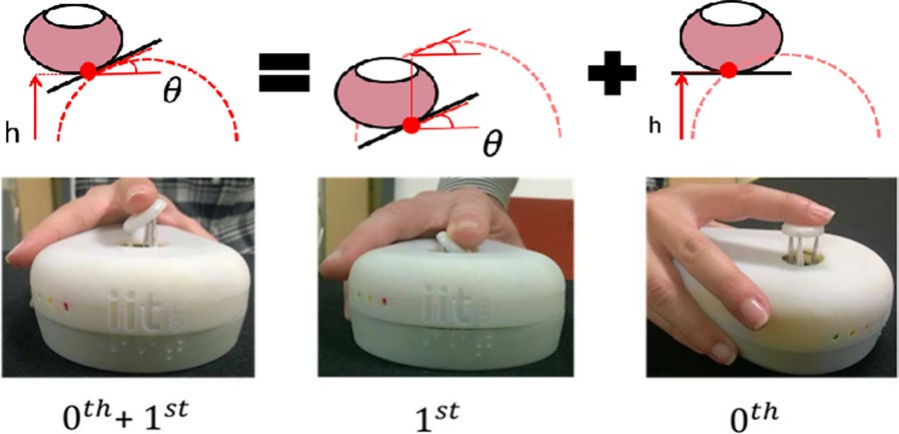
Up: The three geometrical descriptors tested: $0{th}+1{st}$, which is a combination of 1*st* and 0*th* order. Down: The rendering of TAMO3 for each geometrical descriptor

*Procedure* The participants familiarized themselves with the Tactile Dictionary by touching all fifteen solids (sighted participants were blindfolded before entering the experimental room). They perceived virtual objects in three conditions and related sessions, i.e., different tactile modalities (see Fig. 6) associated with a combination of the available geometrical descriptors: elevation alone (0*th* order), inclination alone (1*st* order) or both ($0{th}+1{st}$ order). To avoid possible learning biases, both the object and the order of geometrical descriptors were randomized according to a Latin square design. After the familiarization, one virtual object was displayed: its virtual dimensions matched only the physical dimensions of the *matched size objects* of the Tactile Dictionary. The participants were not aware of this detail. Then, they were requested to explore the object with TAMO3 and to construct the mental map of the object as much accurately as possible. Then they had to indicate which one, among the fifteen real objects, best matched the explored virtual object. No time limit was given. The experimenter recorded the answer on a PC right after it was given, to allow an approximated measure of the exploration time. Each session was composed of 5 familiarization and 15 test trials: each object was presented three times. Each of the 24 participants performed 20 trials in each of the three sessions (total of 1440 trials). After each session, the participants filled the NASA Task Load Index questionnaire (NASA-TLX), a tool to evaluate the participative perception of the workload of a task [54].

*Analysis* We measure participants’ accuracy in matching the shape and size of virtual objects to real objects. We also assessed the mental workload. The independent variables were the *Geometrical Descriptors* (0*th*, 1*st* and $0{th}+1{st}$ order) and the *Group* (VI and BS participants), while the dependent variables were the *Matching Ability* (measured as recognition rate) and the *Mental Workload* (measured with the NASA-TLX).

The errors were classified into two categories: those due to mismatch in size and those due to mismatch in shape. The Matching Ability was defined as the percentage of accuracy in guessing both the size and the shape of the objects. The Appendix reports on the same analyses where only the shape errors (and not size) were considered.

The contribution of size errors was investigated with a separate analysis using the *Size Errors* (the percentage of errors in size with respect to the total amount of trials) as dependent variable and the *Type of Mismatch in Size* as independent variable. The Type of Mismatch in Size was defined as a binary variable to explicit the underestimation and overestimation errors.

On the other hand, the shape errors were classified as a misjudgment of contour (the object borders, i.e., a cylinder confused with an ellipse), of orientation (the position of the object longest side, i.e., a horizontal confused with a vertical object and vice versa) or of both. The errors with the sphere were not included as the object has central symmetry. The appendix (Fig. 16 on the left) describes the three classes of errors with respect to the confusion matrix cells.

To evaluate the quantity of the errors in orientation with respect to those in contour, a ratio was calculated: each class of error was divided for the total number of shape errors.

The Mental Workload was calculating by merging the items of the NASA-TLX questionnaire, weighted by the participants: the complete procedure is described in [55].

Normality and sphericity assumptions of the dependent variables were tested with the Shapiro-Wilk test for within-subjects factors and Mauchly’s test for between-subjects factors, respectively.

Where the analyses were performed on a mixed design and sphericity is confirmed, a repeated measure analysis of covariance (two-factor mixed-design ANCOVA) is reported. In case of sphericity violation, the Greenhouse-Geisser(GGe) corrected the ANCOVA and Bonferroni corrected post-hoc tests are reported. When necessary, an ANCOVA post-hoc (Tukey HSD) analysis was performed. On the other hand, for non-normal distributions, the Friedman Rank Sum test and Wilcoxon non-parametric tests for analysis of variance and post-hoc comparisons were respectively used. In the Figures, starred links join significantly different conditions: *** for p < 0.001, ** for p < 0.01 and * for p < 0.05. Dashed lines indicate a trend, i.e., p < 0.1. R software [56] was used for the analyses.

### Results: ability in matching virtual and real objects

#### Overall results

First, the distributions of matching ability were analysed.

**Fig. 7:**
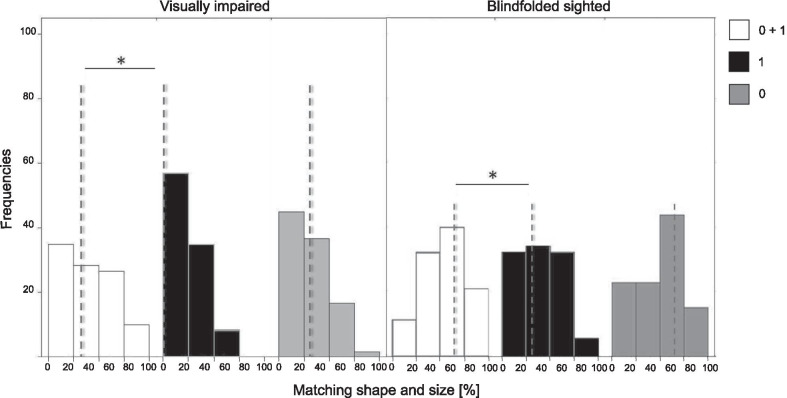
Ability in matching virtual with real objects: histograms have Matching Ability percentages on the x axis (the axis ranges from 0 to 100, spaced by 20%) and the distributions (frequencies) on the y axis. The legend shows the colour code for the *Geometrical Descriptors*. On the left, there are the data of the VI and on the right the data of the BS participants. The dashed grey lines are the median values of each distribution

The ANCOVA investigated for possible modulations of the Matching Ability by Group and by Geometrical Descriptor; it was corrected for the random effect of participants and the nested fixed effect of both factors. It showed a significant effect of both Group [$\hbox {F}(1,22)=16.05$, $\hbox {p}={0.0006}$] and Geometrical Descriptor [$\hbox {F}(2,44)=4.93$, $\hbox {p}={0.011}$]. No interaction effect was observed. The distributions of Matching Ability according to the Group and Geometrical Descriptors are displayed in Fig. 7. Post-hoc t-tests revealed that BS performed significantly better than VI participants [$\hbox {t}(69.97)=4.14$, $\hbox {p}={0.00009}$] and that when both elevation and inclination are present the matching ability is higher than with inclination alone [$\hbox {t}(42.62)=2.67$, $\hbox {p}={0.011}$].

**Fig. 8:**
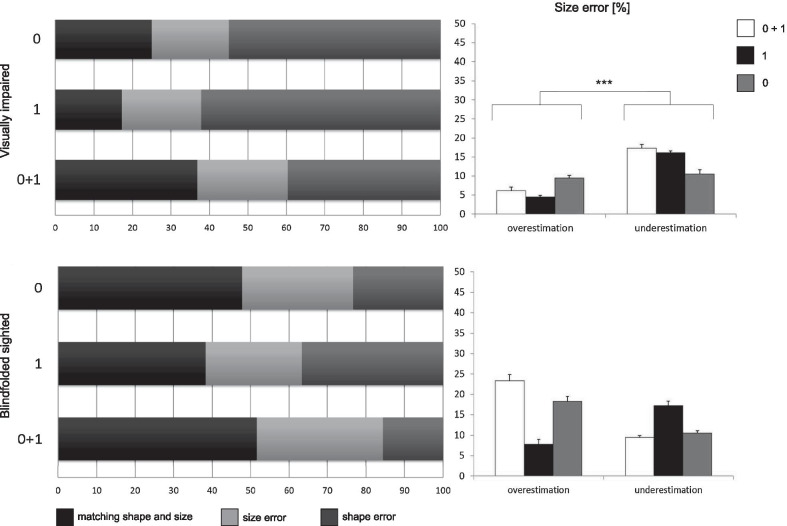
Left: on the horizontal axis there are the percentages of Matching Ability and the errors in size and shape; on the vertical axis there are the geometrical descriptors. Right: the percentages of size errors depending on geometrical descriptors. Percentages are split into overestimation and underestimation errors. Whiskers represent standard deviations from the average values

*Size vs shape errors* We performed a further ANCOVA to investigate possible modulations of the *Size Errors* by Group, Geometrical Descriptors and Type of Mismatch in Size. The ANCOVA was significant only for the Group: BS misunderstood object sizes more than VI participants [$\hbox {F}(1,28.02)=5.96$, $\hbox {p}={0.02}$]. In fact, Fig. 8-left shows an extended light gray area for BS. The Type of Mismatch in Size interacted both with Group [($\hbox {F}(1,70.42)=14.97$, $\hbox {p}={0.0003}$] and Geometrical Descriptor [$\hbox {F}(1,62.50)=13.29$, $\hbox {p}={0.0006}$]. Figure 8-right summarizes these findings. The tendency to overestimate object sizes was more pronounced in BS participants [$\hbox {t}(25.38)=3.32$, $\hbox {p}={0.002}$]. Instead, visually impaired participants underestimated object sizes more frequently. When only inclination was present participants tended to underestimate more often the size of objects [$\hbox {t}(17.99)=4.75$, $\hbox {p}={0.0002}$].

Table 1 shows the values of the shape error ratio depending on the Group and the Geometrical Descriptors. Due to the presence of few data points, we limit our considerations to only qualitative aspects. The contour errors are the most frequent for both Groups, but VI confuse also the orientation more than BS participants.

**Table 1:** Shape error ratio of VI and BS participants

	Contour	Orientation	Contour and orientation	
0 + 1	0.65	0.10	0.24	VI
1	0.52	0.18	0.29	
0	0.58	0.13	0.29	
0 + 1	0.86	0.07	0.07	BS
1	0.89	0.04	0.07	
0	0.86	0.09	0.05	

The following two sections describe the same steps of analysis of the previous section for the VI and BS participants separately.

#### Matching ability within each group

In the VI sample, The ANCOVA test revealed a significant effect of the Geometrical Descriptos on Matching Ability [$\hbox {F}(2,22)=5.01$, $\hbox {p}={0.02}$ corrected with GGe]. Post-hoc t-tests showed that the difference was statistically significant between the $0{th}+1{st}$ and 1*st* condition [$\hbox {t}(15.27)=2.98$, $\hbox {p}={0.01}$]. The conditions in which the elevation cue was presented (i.e., $0{th}+1{st}$ and 0*th*), exhibited the highest values of performances, having both a median value of 33.3% respect to the 0% of $1^{st}$ condition, see Fig. 7 on the left.

Similar results are achieved in the BS sample: Matching Ability was significantly influenced by the Geometrical Descriptors [$\hbox {F}(2,22)=5.23$, $\hbox {p}={0.011}$] and post-hoc showed a statistical difference only between $0{th}+1{st}$ and 1*st* condition [$\hbox {t}(21.22)=1.6$, $\hbox {p}={0.05}$], see Fig. 7 on the right. Both the conditions where the elevation cue was displayed exhibited higher values of performances (median value of 66.7%) than the 1*st* condition (33.3%).

The two groups behaved differently when considering size errors. In all the three tactile conditions, object size was similarly misunderstood by the VI group. ANCOVA revealed that neither overestimation nor underestimation depended on the tactile condition, but there was a underestimation bias in perceiving object size [$\hbox {F}(1,61.63) = 20.97$, $\hbox {p}={0.0001}$]. Underestimation was more often than overestimation [t(23.87), $\hbox {p}={0.0003}$]. See Fig. 8-right-up. No significant difference was found for the BS group (Fig. 8-right-down).

**Fig. 9:**
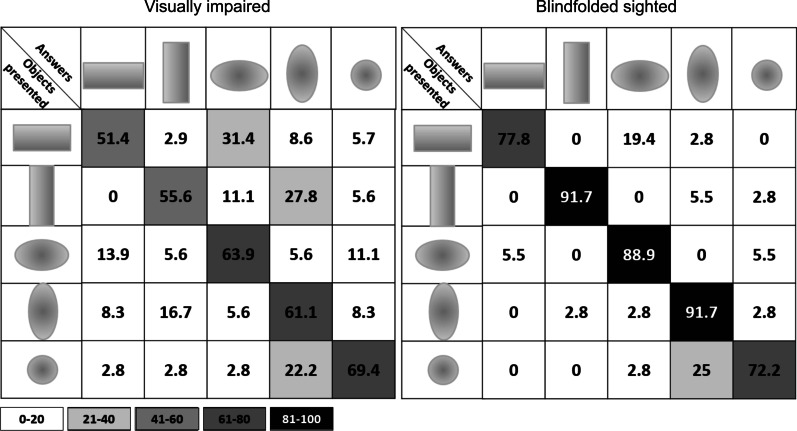
Confusion matrix of performances in matching shapes for the Geometrical Descriptor $0{th}+1{st}$. On the left, there are the data of the VI participants and on the right the data of the BS participants. Cells contain recognition rates: in this case size mismatch was not considered an error

Figure 9 shows the confusion matrix for the condition with the Geometrical Descriptor $0{th}+1{st}$ for both VI and BS: in this case, the size mismatch was not considered an error. For both the VI and BS group, all the values on the diagonal are higher than those outside (VI: p<0.02; BS: p<0.001) meaning that shape is well recognized for all the objects (the accuracy in matching virtual objects, i.e., the F-score, is 60.4 for VI and 84.5 for BS). The confusion matrices of the remaining Geometrical Descriptors are in the Appendix.

### Results: cognitive workload

#### Overall results

A two-way ANCOVA was performed with the Task Load as dependent variable and Group and Geometrical Descriptors as independent variables. ANCOVA was significant only for Geometrical Descriptors [$\hbox {F}(2,44)=8.36$ with $\hbox {p}=$0.0008] and for the interaction between Geometrical Descriptors and Group [$\hbox {F}(2,44)=6.03$ with $\hbox {p}={0.004}$].

Moreover, considering the two samples together, a Post-hoc t-test revealed that condition 1*st* had significantly higher values than 0*th* [$\hbox {t}(41.23)=2.25$, $\hbox {p}={0.02}$] and $0{th}+1{st}$ [$\hbox {t}(42.48)=1.94$, $\hbox {p}={0.05}$] conditions.

#### Cognitive workload within each group

**Fig. 10:**
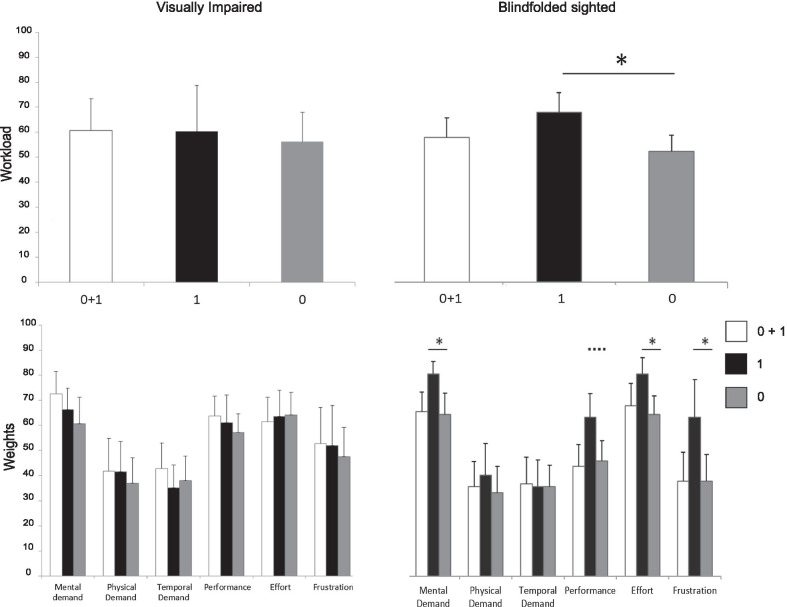
Overall task load values (first row) and their factors (second row) for different tactile feedback. The VI sample is on the left, whereas the BS is on the right. Whiskers represent standard deviations from the average value of factors

The overall workload and their factors are shown in Fig. 10 depending on the Geometrical Descriptor. For the VI group, the perceived workload did not depend on the Geometrical Descriptor. No factor out of six was statistically different, meaning that geometrical descriptors used did not influence the workload associated to the task. Conversely, for the BS group, the Geometrical Descriptor significantly modulated perceived workload [$\hbox {F}(2,20)=5.52$, $\hbox {p}={0.01}$]: its effect is significant between the conditions 0*th* and 1*st* [$\hbox {t}(10)=-5.42$, $\hbox {p}={0.0003}$] having the highest workload in the 1*st* condition. Four out of six factors were statistically different, meaning that geometrical descriptors efficiently differentiate the task. Factors showing a statistical difference were Mental Demand [$\chi ^{2}(2)=8.87$, $\hbox {p}={0.01}$], Performance [$\chi ^{2}(2)=6.68$, $\hbox {p}={0.03}$], Effort [$\chi ^{2}(2)=8.06$, $\hbox {p}={0.01}$] and Frustration [$\chi ^{2}(2)=8.7$, $\hbox {p}={0.01}$]. All the factors listed, statistically differentiated the $0^{th}$ and the 1*st* conditions (mental demand: $\hbox {V}=28$, $\hbox {p}={0.021}$; performance showed a trend: $\hbox {V}=46$, $\hbox {p}={0.065}$; effort: $\hbox {V}=36$, $\hbox {p}={0.014}$; frustration: $\hbox {V}=52$, $\hbox {p}={0.014}$). In agreement with the global workload, the condition 1*st* was generally judged as more mentally demanding, requiring more effort and causing more frustration than $0{th}+1{st}$ and 0*th*.

### Results: performance self-report

**Fig. 11:**
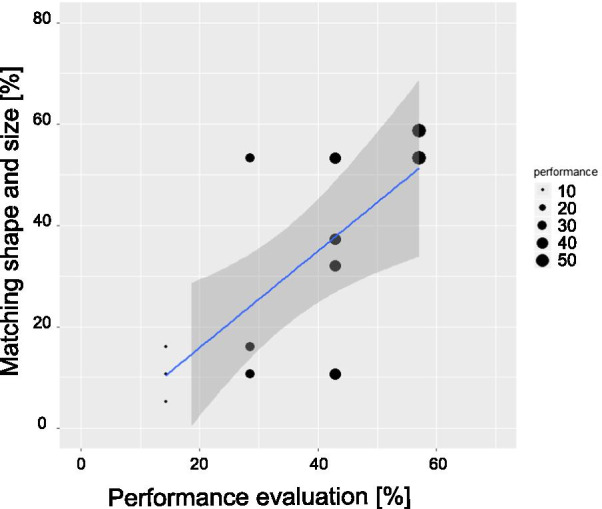
Linear prediction of matching ability from VI participants in the condition $0{th}+1{st}$. Point sizes indicate the amount of values present in that coordinate. The blue line is the straight line predicting matching ability and the grey shaded area represents the standard error

**Fig. 16:**
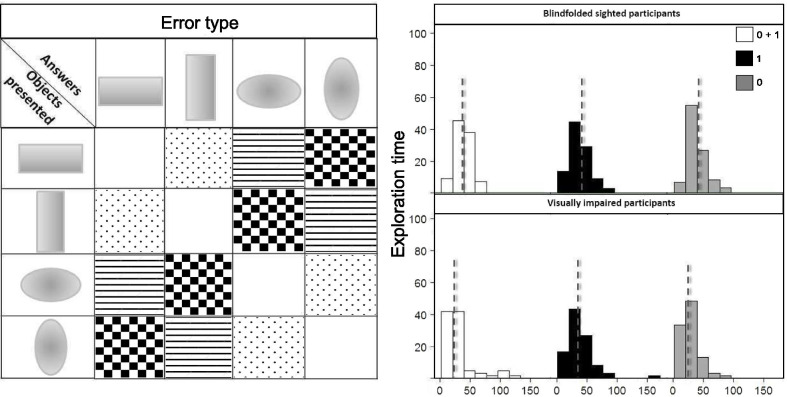
Left: Matrix indicating the three classes of shape errors analysed. Right: Distributions of exploration times depending on the Group and on Geometrical Descriptors. Dashed lines represent mean values

Data about the self-report of the performance, collected with the NASA questionnaire, were compared with the matching ability (where shape and size were considered as errors). Only VI population showed to properly rate the matching ability: a significant correlation was found between the ability to match virtual objects and the subjective evaluation of their performance, $\hbox {F}(1,10) = 13.09$; $\hbox {p}={0.005}$; $R^{2} = 0.57$ (see Fig. 11). This correlation is valid only for the condition in which both Geometrical Descriptors are present. Multiple correlations were conducted with the remaining NASA factors and results confirmed that only self-assessed performance is a reliable predictor (p>0.09).

## Discussion

The way tactile feedback was provided by our experimental setup allowed users to experience local tactile cues, given by elevation and inclination of a tactor in 3DOF, and global kinesthetic cues, given by proprioceptive feedback coming from hand and arm motion. The contributions of the tactile and kinesthetic cues are complementary since they provide information about contours and length respectively. The correct evaluation and integration of both feedbacks foster a better understanding of the virtual scenario. In order to allow a balanced object recognition between the two populations, the virtual objects were created to be unfamiliar 3D shapes [6]. Moreover, for this Tactile Dictionary, curvature discrimination was the main factor to differentiate objects [22]. The smaller curvature of our Tactile Dictionary was chosen from our previous study [57] in order to make sure it was correctly discriminated. The modulation of the tactile feedback alone was introduced to investigate the role of tactile primitives in the ability to match real and virtual objects and their relative cost in terms of mental resources spent. Since curvature discrimination is modulated by the quantity and type of geometrical descriptors depicted, we hypothesized that elevation and inclination cues have different effects. Our results showed that when tactile feedback approximates real touch, performances in matching virtual with real objects are the highest. This result was true for both visually impaired and blindfolded sighted participants. Admittedly, to increase the realism in tactile exploration, the cutaneous feedback needs the implementation of the tactor invariance with respect to the hand rotations (see Sect. 2.4). We cannot exclude that possible misalignments between participants’ expectations and perceptions influenced on the completion of the tasks.

### Merging elevation and inclination cues facilitates object recognition

*Overall results* The use of all geometrical descriptors was a source of good comprehension of virtual objects explored by touch, both in presence of visual impairment or not. However, a difference in accuracy rate was found since visual impairment was associated with lower performance in matching real with virtual objects.

This outcome follows the literature which indicates that exploring objects without manipulating them results in an impaired performance of the visually impaired (VI) population [5]. This was true for both orientation and contour errors. The low accuracy in judging orientation is in line with previous studies demonstrating that haptic orientation discrimination is impaired in absence of vision [58]. One reason for this confusion in estimating the length and width of virtual objects could be explained by the serial nature of haptic exploration [59]. This was true also for blindfolded sighted (BS) participants but to a less extent.

However, BS were less accurate than VI when matching size: they tended to overestimate the dimensions of virtual objects. Dimension misjudgment is a known illusion in the context of active haptic discrimination of 3D physical objects [60]. This effect could be explained by the anisotropic evaluation of shoulders’ angular positions and temporal differences in exploratory movements. To deepen the comparison between the two samples, further analyses regarding the kinematics of the exploratory procedures should be conducted. Moreover, also the tactile feedback polarizes the discrimination of the object sizes. Inclination alone contributes to the perception that virtual objects are shrunk. A possible motivation is in the nature of the cutaneous feedback which is local and does not involve the movement of the entire finger [61].

*Matching ability within each group* For both groups, the condition in which both inclination and elevation are present has a higher matching rate with respect to the performance in which only one geometrical descriptor is present. This suggests that the quantity of tactile descriptors displayed has a positive relation with the ability in matching real with virtual objects. For BS participants, the distributions of matching rates are skewed towards high performances and confusion matrices are mainly diagonal. This is true for the VI group but to a less extent.

**Fig. 17:**
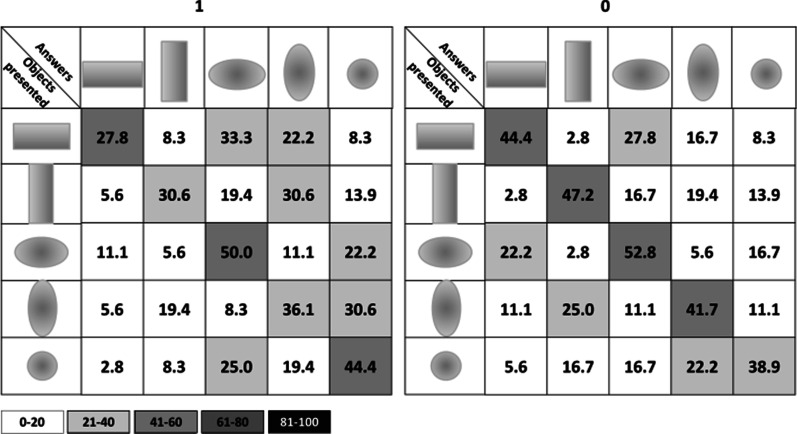
*VI sample*. Confusion matrices of performance of VI participants in matching shapes for the Geometrical Descriptor 1*st* (left) and 0*th* (right). Cells contain recognition rates of matching ability when only shape errors are considered

**Fig. 18:**
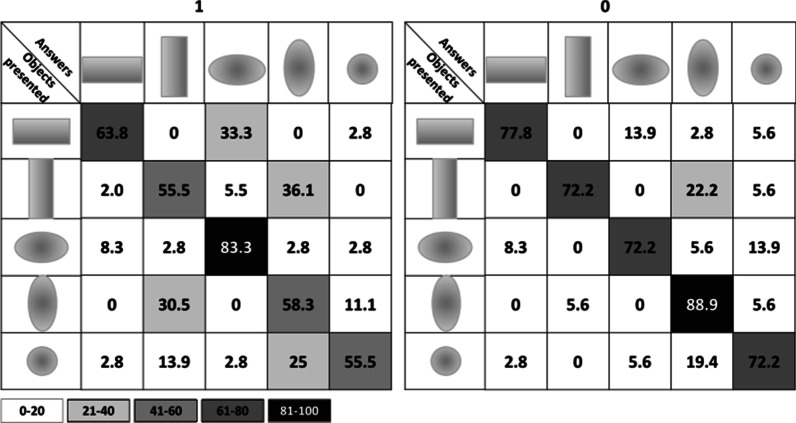
BS sample. Confusion matrices of performance of BS participants in matching shapes for the Geometrical Descriptor 1*st* (left) and 0*th* (right). Cells contain recognition rates of matching ability when only shape errors are considered

The simplification of tactile information led to more confusion when constructing mental representations from virtual objects. If rendered alone, for VI, inclination is not sufficient to elicit a comprehensible mental map of the objects: in fact, the matching rate is lower than the chance level. In the BS group, while inclination cues led to worse performance mainly because of shape errors, when elevation cues are present, size errors are more prominent (see Fig. 8). On the other hand, VI seem to estimate the size independently from the tactile feedback. The main source of error, contour-wise, was the estimation of inclination in more than one dimension: in fact, cylinders were mainly confused with ellipses and vice versa (see the confusion matrices of Fig. 9 and in the “Appendix” 5, Figs. 17,  18). Perceiving different major and minor axes may have led to clearer mental constructions, while an object with central symmetry such as the sphere was frequently confused with an ellipse. This result could be influenced by the hand’s scanning movements and their direction since literature states that haptics judgements are anisotropic [62, 63]. Object orientation was almost always perfectly guessed, especially for BS participants. Proprioceptive cues, leading to estimate orientation, were therefore very well decoded, while tactile cues, leading to estimation of curvatures, were integrated with more issues. This aspect seems to be in contrast with past works showing that inclination cues are sufficient to successfully encode curvature information [24, 57]. A possible explanation might be that perceiving curvatures in a multidimensional space may require more effort. This aspect needs further research. Qualitative observations from our participants reported that when only elevation is displayed, the zones of the objects where the gradient is null (e.g., peaks of spheres, peaks of ellipses and the highest line of the cylinders) could be confused with the zones surrounding the object. This explains the lowest performance achieved in the matching rate.

An evident difference between the groups is the opposite bias in perception of object dimensions. VI appeared to systematically underestimate the size of virtual objects, regardless of the kind and amount of geometrical descriptors displayed. This result has an important consequence if, in a more practical scenario, visually impaired people would independently use the TAMO3. In this case, the size of virtual objects used in the experiment represent the lower limit: passing this limit could penalize object understandability. Interestingly, when BS’s performance is at best, overestimation errors become prominent. Although we do not show the complete confusion matrices (where size errors are also displayed) participants tended to significantly overestimate the dimensions of all five objects. The size estimation seems to be a perceptive bias reported in literature: it is related to the use of visual memory instead of manual memory [64]. However, for practical applications, this bias could be recovered by decreasing the overall size of the virtual objects and allowing a more precise matching.

### Rendered alone, inclination cue increases the perceived workload

*Overall results* Belonging to a group, blindfolded sighted (BS) or visually impaired (VI) participants, does not affect the perception of workload associated with the task. On the other side, only the amount of Geometrical Descriptors makes a difference in evaluating how the task is demanding. Therefore, grouping the two samples, the condition in which there was only the inclination was perceived as the most challenging.

VI population showed particular sensitivity to the evaluation of their performance. Their subjective evaluation clearly reflects the objective accuracy in matching objects: subjective and objective variables can be linked by a linear correlation as already demonstrated in [65]. On the contrary, the BS population failed in this estimate. This is a precious result in perspective of the independently use of TAMO3 by VI people in a learning scenario. In current rehabilitation protocols, VI people are often completely dependent on the teacher and practitioners and passive agents of exercises with fixed environmental constraints. However, it has been shown that the possibility to interact with the tactile environment improves the learning of spatial skills [66].

*Cognitive workload within each group* The kind of geometrical descriptors does not affect the workload perceived by VI participants, even if the tactile condition resembling real touch has statistically higher performances with respect to the other tactile conditions, it is perceived as equally demanding. Surprisingly, the condition verbally rated as the less intuitive, i.e., the one in which only inclination was depicted, was evaluated as challenging as the other. One possible explanation is that the three conditions were equally perceived as abstractions of reality and tactile feedback changes were not sufficient to differentiate those levels of abstractions. On the other hand, the kind of geometrical descriptor influences the perceived workload for BS participants. The highest global workload, as well as the highest mental demand, effort and frustration, are found with inclination cues only. This result matches with the poorest performances achieved in this condition. When displaying elevation only, the task is less mentally demanding and entails less effort, frustration and less global workload. These two observations suggest that simplifying the tactile feedback does not necessarily mean increasing the complexity of the task: what seems to be crucial is the *way* this is done. When both cues are present, the workload sets to an average value (and performance grows at best). The relation between performance and workload is therefore highly task dependent, as also shown in [19].

## Conclusions

In conclusion, this study shows that it is possible to convey information about solid geometry with a portable device delivering limited tactile feedback. This method can be proposed, in an educational scenario, as a complementary learning tool when geometrical concepts have to be displayed by persons with vision loss. The proposed system has the potential to be adapted to the specific skills of the learner. In principle, a learner who is aware of his/her performance, can potentially self-tune task difficulty to challenge more complex tasks. On the same line, a simple software add-on can automatically report on the correctly guessed shapes and help VI learners to repeatedly improve their skills. Ultimately, since digital content can be loaded remotely, a system like TAMO3 can be used at home by one (or more) VI learners. It can serve as a haptic tool for telerehabilitation protocols, where digital content is loaded by a practitioner and spatial skills are daily trained in online or offline game-like sessions. The software can help in collecting performance (matching ability) and behavioural data (mouse motion, time dedicated to exercises) and support the practitioners in deciding on the next steps.

## Figures and Tables

**Fig. 12:**
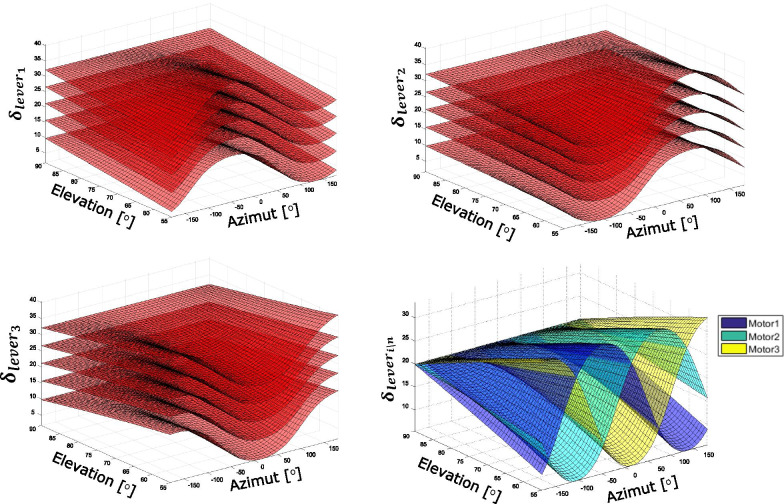
For each value of the heights of the tactor, graphs show simulated values of angles of servo labelled as the first, $\delta _{lever_{1}}$ (top-left graph), of the angles of the second servo, $\delta _{lever_{2}}$ (top-right) and of the angles of the third servo, $\delta _{lever_{3}}$ (down-left), respect to *azimuth* and *elevation* variations. The last graph shows the simulated values of the angles for all the three servos at a specific height: 23.6 mm

**Fig. 13:**
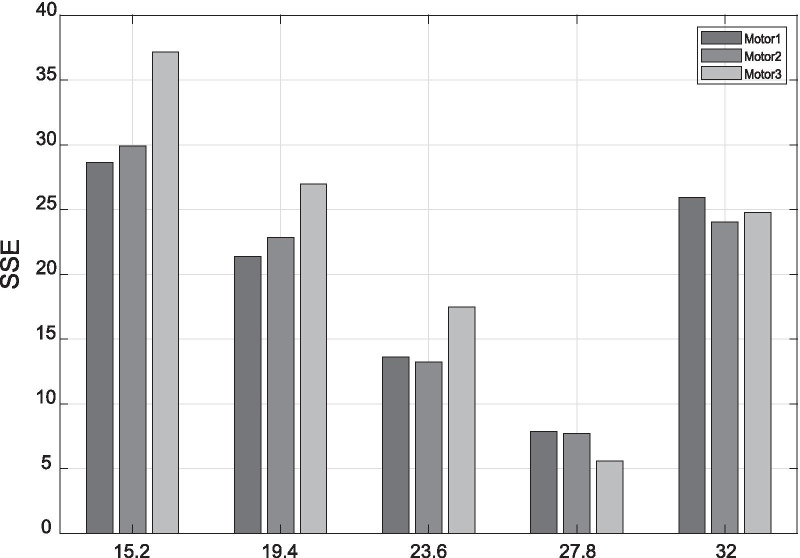
Sum of squares due to errors for the chosen 5 heights of the tactor

**Fig. 14:**
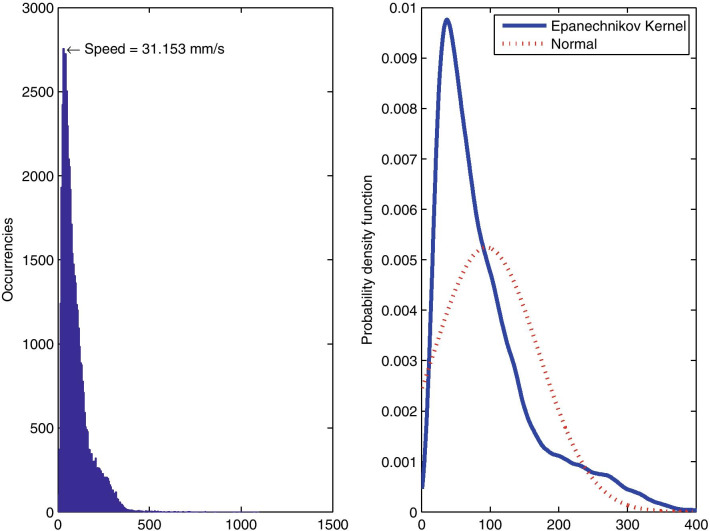
Left: Histogram of the speeds achieved by participants during the experimental tests. Right: density probability distribution of the measured speeds (with maximum value equal to 37 mm/s) and the normal distribution (with the calculated average value equal to 96 mm/s)

**Fig. 15:**
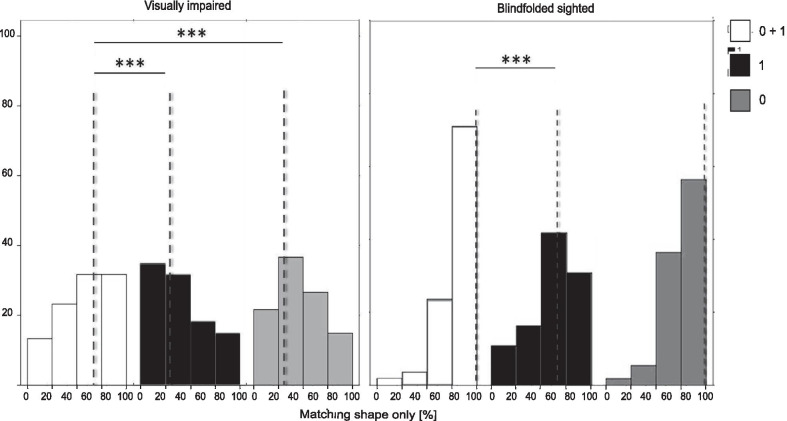
Ability in matching virtual with real objects where the correctness is defined as guessing only shape. Histograms have matching percentages on the x axis and frequencies on the y axis. The legend shows the colour code for each Geometrical Descriptor. On the left, there the VI participants’ data and on the right the BS participants’ ones

## Data Availability

The datasets analysed during the current study are available from the corresponding author on reasonable request.

## Appendix

### Invariance of tactile feedback

To implement the invariance of tactile feedback, TAMO3 is provided with 2 coils: each of them creates a magnetic field transformed in absolute position by a graphic tablet (see Sect. 2.4 for further details about the absolute positioning system). The coils are arranged on the bottom part of the TAMO3 respectively along the same direction of the tactor and where wrist leans. Hand orientation corresponds to the angle of the line connecting the two coils. The method used to update the status of the tactor consists in picking up the data of angles of servos from a lookup table stored in the board of the TAMO3. The choice to store the angles of servos on-board was driven by the need to achieve fast data recall. If those angles were stored in the PC, the time required to access them would be constrained by wireless communication features. Indeed, the communication delay before introducing the lookup table was 58.66 ms. This results from the sum of the time to send the absolute position of the TAMO3 to the PC and the time to send the movement command to the TAMO3 from the PC.

The lookup table was generated for each servo through a simulation performed in Matlab^®^ environment. To decrease the dimensions of the lookup tables and thus the delay to access its data, the simulation was done for a representative set of the heights of the tactor. For simplicity of visualization and calculation, the spherical coordinate system was chosen. The angle of tactor is represented as a function of *azimuth* and *elevation* angles as in the following equation (see Fig. 4):

$$\begin{aligned} \delta _{lever_{s|h}} = f(azimuth,elevation) \end{aligned}$$

where *s* indicates a single servomotor and *h* the set of chosen heights of the tactor, *h* = 15.2,19.4,23.6,27.8,32 mm. In the simulations *azimuth* angle was varied within the range [$-180^\circ$, $180^\circ$] and *elevation* within the range [$55^\circ$, $90^\circ$], results are shown in Fig. 12.

Looking at the plot with the $\delta _{lever_{s}}$ for all the three servos (last plot on the right in Fig. 12) it is evident that, for fixed values of *elevation*, curves have a sinusoidal trend dephased of a certain $\Theta$. For fixed values of *azimuth*, the *elevation* shows a quadratic dependence. Moreover the initial $\delta _{lever_{i|h}}$ status depends on the specific height value. Those considerations led to the formulation of the following model:

$$\begin{aligned} \delta _{lever_{s|h}} =&k_{offset} \\&+(a*Elevation^{2} + b*Elevation + c) * \\&sin(f * Azimuth + \Theta ) \end{aligned}$$

where $k_{offset}$ depends on the height of the tactor and the parameters *a*, *b*, *c*, *f* are the coefficients predicted by the model.

The model is necessary to predict values of $\delta _{lever_{s|h}}$ and successively fill the lookup tables. In Matlab^®^ environment, the model was tested with the functions of the *Curvature Fitting Toolbox*. The chosen algorithm for the fit was the Levenberg-Marquardt method [67, 68], usually implied for least-square estimation in case of non-linear parameters. Figure 13 represents the sum of squares due to errors (SSE), i.e., a statistic that measures the total deviation of the response values from the fit to the actual response values. A low value of SSE indicates that the model has a smaller random error component and that the fit will be more useful for prediction. As depicted in Fig. 13, SSEs have a sinusoidal trend and decrease while increasing the heights of the tactor: at 27.8 mm the SSE are five times less than at 15.2 mm and from 32 mm they increase again. The major source of error is due to the inability of fitting portion of curves with a high slope, thus mostly those next to maximum and minimum peaks. The SSE in fact has its smallest values when the heights of the tactor are next to the boundaries of the working area of servos, i.e., where the available positions to be performed are characterized by small inclinations. However, the discrepancy of SSE should be taken into account while performing perceptual tests and it will be a matter of further evaluations.

### Additional analyses

To confirm the correct display by TAMO3 of the virtual shapes, the participants’ speed was analysed. Figure 14 shows the distribution of averaged speeds achieved during the experimental tests (left) and the density probability distribution of the calculated speeds (right). The range of speeds achieved with the highest number of occurrences is [28.4 , 33.9] mm/s and the average value is 31.2 mm/s. The Epanechnikov kernel was used to estimate the probability density function of the speeds measured since the distribution was not normal. This kernel returns the value of 37 mm/s as most probable value for exploration speed, which is considerably lower than the maximum value of the normal distribution (plotted on the right in red dashed lines) equal to 96 mm/s. The value of the most probable speed, multiplied by the physical delay of motors, i.e., 120 ms to complete the highest movement imposed by shapes dimensions (59.7 deg) returns 4.4 mm which represents the space in which participants are moving but not feeling the correct tactile sensation. This length compared with the maximum dimensions of the shapes, i.e., 100 mm, represents 0.04%. Therefore we can conclude that the adopted device is able to properly render the 2.5D virtual shapes.

In order to compare participants’ speeds and servomotor temporal resolution, table 2 summarizes the main features of servomotors.

**Table 2:** Main features of the servomotor used for TAMO3 device

**Servo HS-5056 MG**	
No-load speed	0.12s/60$^{\circ }$
Stall torque	0.99 kg*cm
Max PWM signal range	900–2100 $\mu$s
Motor type	3-pole
Current drain—idle	3 mA
Current drain—no-load	3 mA
Potentiometer drive	6 slider indirect drive
Gear type	metal

The following paragraphs confirm the main results discussed in the paper using a less rigid definition of matching ability, i.e., only shape mismatch is considered an error.

#### Overall results

The ANCOVA revealed significant effects on both Group [$\hbox {F}(1,22)=13.34$, $\hbox {p}={0.0014}$] and Geometrical Descriptor [$\hbox {F}(1.48,32.62)=11.25$, $\hbox {p}={0.0006}$ corrected with GGe]. Post-hoc t-tests revealed that, when both elevation and inclination are present, the matching ability of the condition with both Geometrical Descriptors is higher than only inclination [$\hbox {t}(23)=4.68$, $\hbox {p}={0.0001}$] or than only elevation [$\hbox {t}(23)=3.68$, $\hbox {p}={0.0012}$] condition. Additionally, BS performed significantly better than VI participants [$\hbox {t}(68.61)=4.86$, $\hbox {p}={0.000007}$], see Fig. 15 .

#### Results within each group

In VI group, ANCOVA revealed a significant effect of the Geometrical Descriptor on Matching Ability [$\hbox {F}(2)=5.58$, $\hbox {p}={0.01}$]. Post-hoc t-tests showed that the performances in $0^{th}+1^{st}$ condition were statistically higher than 1*st* [$\hbox {t}(11)=3.31$, $\hbox {p}={0.007}$] and 0*th* [$\hbox {t}(11)=3.14$, $\hbox {p}={0.009}$] condition. In this case performances of $0{th}+1{st}$ and $0^{th}$ are higher than 1*st*, showing a median of 66.7% for $0{th}+1{st}$ and median of 33.3% for the remaining tactile conditions. Therefore, the $0{th}+1{st}$ and 1*st* conditions were different when correctly guessing both shape and size or shape alone. See Fig. 15-left for more details.

Similarly in BS group, ANCOVA showed a significant effect of the Geometrical Descriptors on Matching Ability [$\hbox {F}(2)=3.99$, $\hbox {p}={0.03}$]. Post-hoc t-tests revealed that the difference was statistically significant between the $0{th}+1{st}$ and 1*st* condition [$\hbox {t}(11)=3.17$, $\hbox {p}={0.009}$]. Performances of $0{th}+1{st}$ and 0*th* are higher than 1*st*, showing a median value of 100% for both conditions in which the elevation is present. See Fig. 15-right for more details.

In Fig. 16, on the left, are shown the three classes of errors that have been analysed. Polka dot texture indicates the errors in orientation, i.e., a horizontal confused with a vertical object and vice versa. Horizontal line texture represents the errors in misjudging object shape, i.e., a cylinder confused with an ellipse with the exact orientation. Chess-board texture defines the shape and orientation errors, i.e., a horizontal cylinder confused with a vertical ellipse. White coloured cells indicate an exact matching.

Figure 17 shows the confusion matrices of the VI group, for the Geometrical Descriptor 0*th* and 1*st*. In 1*st* condition, values of performance on the diagonal (mean value is 37.8%) are statistically similar to those outside (p>0.05): horizontal and vertical ellipses are confused with spheres and vice versa; horizontal cylinders are mainly confused with horizontal ellipses and vice versa; vertical cylinders are confused with vertical ellipses. In the 0*th* condition, the prevalence of correct values on the diagonal does not occur (p>0.05), although the confusion with objects of similar orientation is still apparent. In practice, a chess-like appearance denotes that confusion occurs with objects having similar orientation with respect to the body of the participant. Overall on the three confusion matrices (considering also the one in Fig. 9-left), ellipses appear to have been better recognized on average (vertical: 46.4%, horizontal: 55.6%), followed by cylinders (vertical: 44.5%, horizontal: 41.2%) and spheres (50.9%) which are mainly confused with ellipses.

Figure 18 shows the confusion matrices of the BS group, for the Geometrical Descriptor 0*th* and 1*st*. In $1^{st}$ condition, values of performance on the diagonal (mean value is 63.3%) are still higher than those outside (p<0.05) but the difference is less evident: specifically, horizontal ellipses are confused with horizontal cylinders; vertical ellipses are confused with vertical cylinders and vice versa. In the $0^{th}$ condition, the prevalence of correct values on the diagonal still occurs (p<0.001), although the confusion with objects of similar orientation is still apparent. Therefore, BS participants share a similar chess-like pattern in the confusion matrices. Overall on the three confusion matrices (considering also the one in Fig. 9-right), ellipses appear to have been better recognized on average (vertical: 79.6%, horizontal: 81.5%), followed by cylinders (73.1% both for vertical and horizontal) and spheres (66.6%) which are mainly confused with vertical ellipses.

### Exploration time

The exploration time did not differ statistically speaking. The exploration time distributions according to the Group and the Geometrical Descriptors are reported in Fig. 16 (on the right). BS appeared to be slower than VI participants. This is confirmed also when the exploration times were divided according to the Geometrical Descriptor.

## Abbreviations

DOF: Degree of freedom
VI: Visually impaired
BS: Blindfolded sighted
